# For a new world ranking of countries in elite sport—correlation between competition level and bibliometrics in Olympic sports

**DOI:** 10.3389/fspor.2025.1489652

**Published:** 2025-08-20

**Authors:** Nadim Nassif, Grégoire Millet

**Affiliations:** ^1^ Physical Education and Sports, Notre Dame University-Louaize (NDU), Zouk Mosbeh, Lebanon; ^2^ Exercise Physiology at the Institute of Sport Sciences, University of Lausanne, Lausanne, Switzerland

**Keywords:** olympics, sports, ranking, bibliometrics, scientific

## Abstract

**Introduction:**

The Olympics remain today the largest multidisciplinary sports competition in the world, and the Olympic Medal Table has been dogmatically followed by media, scholars, and sports administrators, as the mainstream measurement of the countries’ success in elite sport.

**Methods:**

Since 2018, the Olympic Medal Table has been challenged by a global sport scientific index, the World Ranking of Countries in Elite Sport (WRCES), which provided, for the first time, a research-based measurement of the performance of all the countries having National Olympic Committees. The main characteristics of the WRCES is a weighting of each sport determining its level of competition. This coefficient is the sum of two variables, one related to the universality and the other to the global media popularity of each sport. In this paper, correlations calculi, using Pearson R coefficient of correlation along with the corresponding *P* value, will be conducted between the number of citations, number of articles, WRCES level of competition and the number of medals of each Olympic sport.

**Results:**

There is a strong correlation between the number of scientific citations (R = 0.74; *p* < 0.001) or articles (R = 0.70; *p* < 0.001) and the competition level determined by the WRCES while no relationship was found with the number of medals available at the Summer Olympics.

**Discussion:**

Overall, the present study confirms the relevance of the WRCES and adds an argument to contest the rationality of the Olympic Medal program.

## Introduction

Although according to the Olympic Charter, the International Olympic Committee (IOC) and the local organizing committees of the Olympic Games shall not draw up any global ranking per country (1), media, scholars and sports administrators have traditionally referred to the Olympic Medal Table (OMT) as a benchmark to measure the countries performances in international competitions (2). The OMT's validity was first contested by Nassif (3), who identified its following main limitations:

1. The non-consideration of the level of competition of each sport.
2. The low number of countries that win medals.

To address these shortcomings, Nassif (3) has created the World Ranking of Countries in Elite Sport (WRCES), which is characterized by a computation model that allows all the countries to be listed, and the introduction of coefficients that are scaled according to the level of competition of each sport. These two criteria are the main assets of the WRCES, which, to date, is the only research-based index measuring the countries performances in international sport (4). The ranking of all the countries allowed the identification of the framework of factors that lead to the countries’ success in elite sport, which was published in a book in 2023 (5).

This paper will focus on the second major aspect of the WRCES, which is the level of competition, a coefficient that Nassif (3) has calculated by doing the sum of the universality and global media popularity of each sport. This aspect of the WRCES will be compared to the bibliometric analysis weighting the “interest of the scientific community for a sport” (i.e., the “scientific weight”) that was performed for all Summer and Winter Olympic sports by Millet & al (6). The objective of this paper is to investigate whether the WRCES’ level of competition of each sport is related to its bibliometrics size. If there is indeed a correlation between the WRCES’ level of competition and the scientific weight of each Olympic sport, it will further elevate the WRCES as a more accurate and comprehensive ranking for countries in elite sport and benchmark it as a unique index that all the National Olympic Committees (NOCs) could refer to when they want to evaluate their national sports policies.

In the Olympic Charter, the Article 57 (1) titled “Roll of honour” stipulates that “the IOC and the OCOG (Organizing Committees of the Olympic Games) shall not draw up any global ranking per country”. Despite this fact, the OMT, whose methodology results from media interpretation, is highlighted in almost all of the international press at each edition of the summer and winter Olympic Games. The global media attention raised by this ranking pushed even the IOC to present it on its official website, contrary to the principles of the Olympic Charter (7).

There are two interpretations of the OMT, the first, which is the most widespread worldwide (3), which prioritizes the number of gold medals, and the second, used mainly by the US media (8), which prioritizes the total number of medals [(9), p.48]. These two rankings are actually highlighted on the IOC's website (10).

In the interpretation that prioritizes the gold medals, the OMT computes the gold, silver, and bronze medals obtained by different countries in different sport events, in every edition of the Summer and Winter Olympic Games. A gold has superior value over any number of silver, and a silver has superior value over any number of bronze. In the event where two countries obtain the same number of gold, the country with more silver is better ranked. In the case where two countries obtain the same number of gold and silver, the country with more bronze would be better ranked.

In the interpretation that ranks countries according to the total number of medals won, medals are counted regardless of their colors. In the event that two countries obtain the same total number of medals, the one with the most gold medals will be ranked higher. In case two countries obtain the same total number of medals and gold medals, the country with the most silver medals will be ranked higher. In case two countries obtain the same total number of medals, gold and silver medals, the country with the most bronze will be ranked higher. For De Bosscher [(9), p.48], “this measurement wants to avoid the absolute superiority of a gold medal over any number of silver and of a silver over any number of bronzes”. However, for De Bosscher [(9), p.49], this system “does not take into account the relative value of medals”.

De Bosscher [(9), p.49] has given an account about another system that awards points for the medals, three for the gold, two for silver and one for bronze. This pointing system is actually a compromise between the absolute performance and the total number of medals won. De Bosscher [(9), p.50] has then proposed to do a system of “market share”, which consists of the “points won as a proportion of points available to win”. She advocated that this system is the best combination of the absolute ranking, the total medal count, and the pointing system, and therefore an accurate measurement of countries’ performances in the Olympics.

An alternative system has also been promoted by the media to count the medals, the “medals per capita”, which divides the number of medals by the population (11–13). This methodology was strongly questioned by several scholars. Bernard and Busse (14) explained that “first, countries cannot send athletes in proportion to their population for each event where each country is determined a number of athletes by the IOC in negotiation with the country's Olympic committee. As a result, not all the Olympic caliber athletes from a large country are able to participate”. This was also advocated by Den Butter and Van Der Tak (15): “A country with two times as many inhabitants as another country is not expected to win two times as many Olympic medals.. This may partly be caused by the fact that each country is only allowed to delegate a limited number of participants per sport events”. By giving the examples of India and Bangladesh, two of the most populated countries of the world, which had very little or no success (Bangladesh never won a medal in the Games) in the Olympics, Kuper and Sterken (16) showed that population is not an asset for success. For Bian (17), a large population can even worsen countries’ performances, because adding more athletes will reduce the funding of each of them and deteriorate their training conditions.

To date, the most widespread media interpretation of the OMT is the one prioritizing the absolute superiority of the gold medal. Despite its global popularity, Nassif (3) has found that the absolute superiority of the gold medal creates a false inference that places a country having one exceptional athlete capable of winning a gold medal in front another which did not get a gold medal, but was endowed with several athletes who were placed second and third. Another issue identified by Nassif (3) is that the number of medals awarded per event does not take into account neither the level of competition of the sport to which it belongs nor the number of countries and athletes it involves. For example, a sport like sailing that has 10 events and is played in 115 countries offers 10 gold medals, whereas a sport like basketball that has only two events and is played in more than 200 countries. Moreover, for the same event, sailing as an individual sport can offer medals to several athletes from the same country, whereas basketball as a team sport can only offer one medal per country. In that sense, following the Olympic medal table methodology, a minor sport could largely outweigh a major sport that is more popular, universal, and thus more competitive. This has created a false idea of superiority for certain countries which have invested on sports where rivalry is low, and where they could have a competitive advantage (18).

The third major issue that Nassif (3) has identified in the OMT is the low number of countries winning medals, which represent less than 50% of the NOCs participating. For Henry et al. (19), this fact is one of the main weaknesses of the OMT.

It is with the aim of including all the countries in an index that will consider the level of competition of each sport that Nassif has created the WRCES (2018), which methodology will be explained in the next part of this paper.

## Materials and methods

### Overview of the sports level of competition identified by the WRCES

In the WRCES, two variables, universality and global media popularity, were chosen to determine the level of competition of each sport. Universality was considered because it takes into account the number of all the countries participating in a sport. The more countries participate, the more difficult it is for each to win. As for global media popularity, it indicates the international media ratings for each sport. These data show to which extent a sport is covered, and consequently, how much this sport attracts private and public funding and raises the competition level by engaging the most talented athletes (3).

The starting point of the WRCES is a weighted pointing system that replaces the three-medal one in any event, discipline, or sport, which are defined by the IOC glossary as follows (see Table 1).

**Table 1:** IOC glossary for sport, discipline, and event (10).

Term	Definition	Example
Sport	A group of disciplines or events that belong to the same international federation	Aquatics
Discipline	A branch in a sport comprising one or more events	Swimming is a discipline in the sport of aquatics.
Event	A competition in a **sport** or **discipline** that gives rise to a ranking	Men 50 m freestyle is an **event** in the **discipline** of swimming that belongs to the **sport** of aquatics.

Since the number of NOCs that participate in the international Olympic movement is 206, any team or athlete participating in an event, whether a team or individual sport, is granted a basic score of 206, the second is granted 205, the third, 204, and so on. To reward the top eight participants in every event, a weighting coefficient inspired by the Formula 1 scores was introduced as such: The winner of the event will have their basic points multiplied by ten, the second by eight, the third by six, the fourth by five, the fifth by four, the sixth by three, the second by two, and the eighth by one. All those ranked eighth and above will obtain points that decrease from 199 to 1, as it is explained in Table 2.

**Table 2:** Points classification within an event, discipline, or sport (3).

Rank in an event, discipline, or sport	Basic number points (granted on the basis of the number of NOCs)	Weight (Formula 1 2003–2009 scale)	Weighted basic number of points
1	206	10	2,060
2	205	8	1,640
3	204	6	1,224
4	203	5	1,015
5	202	4	808
6	201	3	603
7	200	2	400
8	199	1	199
9	198	1	198
10	197	1	197
11	196	1	196
……	……	……	……
……	……	……	……
……	……	……	……
……	……	……	……
……	……	……	……
206	1	1	1

This pointing system has been used since 2014 and was adopted in 2023 by the International Center for Sport Policy & Governance (ICSPG), a think tank hosted by Notre Dame University-Louaize (NDU), Lebanon (4). Starting 2024, the points obtained by the athlete ranked 8th were multiplied by 1.5. This decision was taken by the ICSPG's team to reduce the gap between the athletes or teams ranked 7th and 8th. Therefore, starting from 2024, the athlete or team ranked 8th will be awarded 298.5 points instead of 199.

In case there are more than 206 athletes in an individual sport event, countries that have no athlete ranked in the top 206 will still have points if the top 206 athletes are from less than 206 countries. These countries will be ranked below the last ranked country that has one of its athletes in the top 206. The more they have athletes ranked after 206, the better ranking they will have. This was done to make sure that every country participating in an event, discipline or sport will get points.

If a sport has several disciplines, the points won in every event are computed by discipline (see Table 3), and the points won in every discipline are computed by sport (see Table 4) following the same pointing system explained above. If a sport does not have any discipline, the points won in every event will be computed by sport (see Table 5).

**Table 3:** Sample of disciplines in which the “summing-up rule” of events is being applied.

Disciplines examples	Points
Swimming	The sum of the points gained in the different men's and women's swimming events (e.g.,100 m freestyle men, 100 m freestyle women, 200 m butterfly men, 400 m freestyle relay, etc.) for country rankings
Water polo	The sum of the points gained in the men's and women's water polo events for country rankings

**Table 4:** Sample of sports in which the “summing-up rule” of disciplines is being applied.

Sports examples	Points
Aquatics	The sum of the points gained in the different aquatics’ disciplines: diving, swimming, synchronized swimming, and water polo for country rankings
Equestrian	The sum of the points gained in the different equestrian disciplines: dressage, eventing, jumping, etc., for country rankings

**Table 5:** Sample of sports in which the “summing-up rule” of events in sports without different disciplines is being applied.

Sports examples	Points
Athletics	The sum of the points gained in the different men's and women's athletics events (pole vault, long jump, high jump, triple jump, 100M, marathon, etc.) for country rankings
Rowing	The sum of the points gained in the different men's and women's rowing events (single sculls men, pair women, eight men, etc.) for country rankings

The points won in the ranking of each sport are then multiplied by the coefficients of universality and media popularity. These coefficients are first used to weigh some of the events witnessing a large difference between men and women, whether superiority is accorded to men or women. The points gained in the different men's and women's events will be multiplied by these coefficients before calculating their sum that leads to the rankings of disciplines. In sports where a difference between men and women is not spotted, the events will not be weighted.

Points gained in the different disciplines will be multiplied by their universality and popularity coefficients before calculating their sum, which results in sports rankings. The points won in the different sports will be multiplied by their universality and popularity coefficients.

### How is the universality coefficient calculated?

As explained in Table 6, the universality coefficient is calculated based on the sport's number of national federations, its presence in the programs of the Olympics, the International School Sport Federation (ISF), International University Sports Federation (FISU), International Military Sports Council (CISM), International Police Sport Union (USIP), International Masters Games Association (IMGA), World Transplant Games Federation (WTGF), Special Olympics (SO), the International Committee of Sports for the Deaf (CISS), the International Workers and Amateurs in Sports Confederation (CSIT), and the Committee of the International Children's Games (CICG), all international multisport organizations recognized by the IOC and having a determined official program.

**Table 6:** Example of the attribution of the universality coefficients in the 2019 WRCES.

Coefficient values	Athletics
Nf% = Number of federations/100	2.06
Olympics program coeff. = (Number of federations/100)	2.06
ISF program coeff. = min (116; the number of national Schools sports federations)/100 (116 being the max number of national Schools sports federations)	1.16
FISU program coeff. = min (173; the number of national University sports federations)/100. (173 being the max number of national University sports federations)	1.73
CISM program coeff. = min (138; the number of national Military sports federations)/100. (138 being the max number of national Military sports federations)	1.38
IMGA program coeff. = min (100; the number of national Master sports federations)/100 (100 being the max number of national Master sports federations)	1
WTGF program coeff. = min (59; the number of national Transplant Games sports federations)/100 (59 being the max number of national Transplant Games sports federations)	0.59
SO program coeff. = min (172; the number of national Special Olympics federations)/100 (172 being the max number of national Special Olympics sports federations)	1.72
CISS program coeff. = min (113; the number of national Deaflympics sports federations)/100 (113 being the max number of National Deaflympics sports federations)	1.13
CICG coeff. = min (32; the number of national Children's Games sports federations)/100 (32 being the max number of national Children's Games sports federations)	0.32
CSIT coeff. = min (30; the number of national Workers and Amateurs sports federations)/100 (30 being the max number of national Workers and Amateurs sports federations)	0.3
USIP coeff. = min (71; the number of national Police sports federations)/(71 being the max number of national Police sports federations)	0.71
**Total universality**	**14**.**17**

If within a sport, a difference in terms of universality between the different disciplines is noticed, there will be a difference in the universality coefficient between them. Since they are run by the same international federation, however, the universality coefficient of the sport will be equal to the discipline that has the highest universality coefficient.

### How is the popularity coefficient calculated?

The first step is to measure the presence of the different sports on each country's top sports website in a one-year span. The top sports website of each country was selected to make sure that the sports appearing regularly in this domain are the most popular in the related country. To identify the top sports website of each country, we used the web portal “Alexa”, which gives accurate data on the top website by industry in each country (20)^1^. Table 7 will show the top sport website in each of the 20 world's wealthiest countries:

**Table 7:** 2021's list of the top sport websites in the 20 world's wealthiest countries.

Wealthiest countries	Top sport website
USA	ESPN
China	China Daily
Japan	Mainichi
Germany	Bild
India	Times of India
United Kingdom	The Guardian
France	“L’Equipe”
Italy	“La Gazetta Dello Sport”
Canada	TSN
Russia	Rsport
South Korea	sports.daum.net
Brazil	Globo
Australia	News
Spain	Marca
Mexico	“El Universal”
Indonesia	“Tribunsport”
Netherlands	nu.nl/sport
Saudia Arabia	“Kooora”
Türkiye	“Ensonhaber”
Switzerland	20 min

The more a sport has weekly news featured on each identified website, the more popular this sport will be in each country. The most popular sport event will receive a score of 100, and those that are ranked below will receive points according to the rule of three. As an example, if a country has eight popular sports events, the most popular one will get 100 points, and the seven others will get the following:

(Points for the 2nd most popular sport event * 100)/8 = (7*100)/8 = 87.5
(Points for the 3rd most popular sport event * 100)/8 = (6*100)/8 = 75
(Points for the 4th most popular sport event * 100)/8 = (5*100)/8 = 62.5
(Points for the 5th most popular sport event * 100)/8 = (4*100)/8 = 50
(Points for the 6th most popular sport event * 100)/8 = (3*100)/8 = 37.5
(Points for the 7th most popular sport event * 100)/8 = (2*100)/8 = 25
(Points for the 8th most popular sport event * 100)/8 = (1*100)/8 = 12.5

These points will then be multiplied by a coefficient based on the GDP of each country. The multiplication of the points by a country's GDP coefficient was done because Nassif (3) considered that a sport that is popular in wealthy countries attracts more funding, leading to the involvement of a higher number of talented athletes, which will raise the level of competition. Here, every trillion dollars gives one point to the GDP coefficient. Given that, for example, France's GDP is 2.58 trillion, the French GDP coefficient will be 2.58. Therefore, the most popular sport event in France will have 258 points. The popularity points won by a sport event in each country are then added to get their total number of points in the world. The most popular sport event in the world will have a popularity coefficient equal to the total number of sports. For example, in 2019, 112 sports were included (37)^2^. Men's football, being the most popular sport event, got 112. The popularity coefficient of the different sports (that of their most popular sport event) was calculated according to the rule of three. As an example:

(Men's basketball * 112)/Men's football total popularity points = (5537.56*112)/7042.887 = 88.06

All the sports will receive a first sub-coefficient based on this rule of three.

The second sub-coefficient will be based on the number of countries where this sport event is popular. Here again, the sport that will be present in the largest number of top countries’ sports websites will receive a second sub-coefficient equal to the total number of sports, and all the other sports will get a second sub-coefficient based on the rule of three. As an example:

(Men's basketball number of countries where it is popular* 112)/Men's football number of countries where it is popular = (151*112)/194 = 87.17

The final popularity coefficient of each sport event will be the average of the first and second sub-coefficients. The final popularity coefficient of each sport will be equal to the coefficient of its most popular event. As an example, men's basketball being the most popular basketball event, basketball will have the following popularity coefficient:

(Men's basketball first popularity sub-coefficient + Men's basketball second popularity sub-coefficient)/2 = (88.06 + 87.17)/2 = 87.6

The final coefficient of each sport, which is equal to the sum of the universality and global media popularity coefficients, will determine the level of competition of each sport. Table 8 will show how the level of competition was calculated for all the Olympic sports in 2021:

**Table 8:** Calculation of the level of competition coefficients of the different Olympic sports for the year 2021 according to the WRCES methodology.

List of the olympic sports	Media popularity coefficient	Universality coefficient	Level of competition coefficient (the sum of the media popularity and universality coefficients)
Football	112	12	124
Basketball	87.6	11	98.6
Tennis	54	8	62
Athletics	36	15	51
Volleyball	33	14	47
Boxing	38	8	46
Golf	35	10	45
Ice Hockey	34.1	5.2	39.3
Baseball/Softball	33	6	39
Aquatics	22	14	36
Cycling	22	13	35
Handball	25	5	30
Table Tennis	15	12	27
Judo	12	13	25
Badminton	13.4	11.5	24.9
Wrestling	15	9	24
Weightlifting	14	6	20
Skiing	8	11	19
Shooting	10	8	18
Gymnastics	9	8	17
Fencing	9	7.5	16.5
Archery	7.8	8.6	16.4
Rugby Sevens	15.1	0.6	15.7
Taekwondo	5.39	10.05	15.4
Sailing	11	4	15
Skating	9	5	14
Karate	5	8	13
Rowing	9.2	3	12.2
Triathlon	6.3	5.8	12.1
Field Hockey	11	1	12
Equestrian	4.5	7	11.5
Curling	6.2	4.4	10.6
Biathlon	6.6	3.9	10.5
Canoe-Kayak	6	3	9.3
Modern Pentathlon	5	2	6.7
Surfing	3.2	1.5	4.7
Bobsleigh	3	1.4	4.4
Luge	3	1	4
Skateboarding	2	1	3
Sport Climbing	0.4	1.8	2.2

The WRCES allowed an assessment of the performances of all the countries having NOCs, a consideration of the level of competition of each sport, and the possibility of identifying the factors that allow a country to succeed in elite sport, work which was published in a scientific book in 2023 (2). Correlations between the 2014, 2015, 2016, 2017, 2018, and 2019 editions of the WRCES were made with the population, GDP, GDP per capita, area, population density, and research output rankings of the same countries for the six corresponding years. These calculi have showed that GDP and research output have the strongest impact on countries’ results in elite sport, with research output, with an average correlation of 0.8, exceeded even the one of the GDP, which stood at 0.76 (5). This high incidence of research on sport performance witnesses the congruence between science and sport, which will be further explored in the next part of this paper.

### Overview of the sports’ bibliometrics presented by Millet et al. (6)

Sports’ bibliometric has already been the subject of extensive academic research. Hanief (21) has undertaken a bibliometric analysis to analyze to trends of publications in the scientific review “Journal Sport Area”. Gholampour et al. (22) did a similar study for the journal “Sport Management Review”. Lòpez-Carril et al. (23) undertook a bibliometric analysis on the rise of social media in sport. Escher (24) did a bibliometric study on the sustainable development in sport. Ciomaga (25) undertook a holistic bibliometric analysis on the central themes and trends in sport management. Baier-Fuentes et al. (26) studied the bibliometric related to the emotions in sport management. Also, in the field of sport management, Shilbury (27) focused on the bibliometrics of four academic journals: “Journal of Sport Management”, “Sport Marketing Quarterly”, “Sport Management Quarterly”, and “Sport Management Review”. Belfiore et al. (28) did a bibliometric analysis on sport for health promotion. González-Serrano et al. (29) undertook a bibliometric analysis to give an overview of the sport entrepreneurship field. Through a bibliographic search of the Web of Science, PubMed, and Google Scholars databases, Pilon and Prince (30) have shown there is a strong link between scientific productivity and the number of medals won for the 2012–2022 Paralympic Games.

In this paper, we will focus on the bibliometric research done by Millet et al. (6), considering that it is the only one that presented a comprehensive overview on the Summer and Winter Olympic sports. This study will allow us to do a correlation calculi between the Olympic sports’ bibliometrics and their level of competition. How was this research conducted?

Millet et al. (6) first gathered the data by proceeding with a search of 116 sports science journals in the web portals PubMed and Web of Science. They then expanded their search to other journals having the terms “exercise” or “sport” in their titles. As a second step, they limited their research to sports that are part of the Tokyo 2020 and Beijing 2022 Olympic programs. For each sport, the articles were listed based on citation frequency from highest to lowest, and the main metrics were averaged for the top 10 articles in each sport. They also compiled the keywords related to six major research topics in each sport: physiology, performance, training and testing, injuries and medicine, biomechanics, and psychology. Following this, they recorded the 5 most cited articles for every summer and winter Olympic sport.

### How were the correlations calculated between the sports’ bibliometrics and level of competition?

Normal distribution of the data was checked by the Shapiro–Wilk normality test. Correlations between the number of citations, number of articles, WRCES level of competition and the number of medals at the Olympics were calculated using Pearson R coefficient of correlation along with the corresponding *P* value. Significance was set at *P* = 0.05. All analyses were completed using SigmaStat (version 3.5; Systat Software®, San Jose, CA).

## Results

As stated in the introduction, the objective of this paper was to compare the level of competition in Olympic sports^3^ with their bibliometrics, in view of contesting the validity of the OMT. Since the research of Millet et al. (6) targeted the sports that were present in the 2020 summer and 2022 winter Olympics, which were organized in a 7-month span between August 2021 and February 2022, the bibliometrics will be compared to the WRCES 2021 methodology. In their study, Millet et al. (6) searched 116 sport/exercise journals on PubMed for both the summer and winter sports. 34,038 articles were filtered for a final selection of 25,003 articles (23,334 on summer sports and 1,669 on winter sports) and a total of 599,820 citations. For football, Millet et al. (6) have also counted the number of citations and publications every time the sport was referred to as “soccer”. Table 9 will show the level of competition coefficients of the Olympic sports in 2021 according to the WRCES, in comparison with the number of citations, scientific papers, and medals in the Olympics given for each country.

**Table 9:** Level of competition coefficients of the different Olympic sports according to the WRCES 2021 methodology, compared to the total number of citations and the number of medals offered.

List of the olympic sports according to their level of competition (points)	List of the olympic sports according to their number of citations	List of the olympic sports according to their number of scientific papers	List of the olympic sports according to the number of medals offered for countries
1- Football (124)	1- Football (157,646)	1- Football (4,937)	1- Skiing (55)
2- Basketball (99)	2- Cycling (87,257)	2- Cycling (3,614)	2- Aquatics (49)
3- Tennis (62)	3- Athletics (85,449)	3- Athletics (3,404)	3- Athletics (48)
4- Athletics (51)	4- Aquatics (42,909)	4- Aquatics (2,197)	4- Skating (28)
5- Volleyball (47)	5- Basketball (28,420)	5- Baseball/Softball (1,071)	5- Cycling (22)
6- Boxing (46)	6- Baseball/Softball (25,146)	6- Basketball (1,042)	6- Gymnastics (18)
7- Golf (45)	7- Tennis (22,791)	7- Tennis (954)	6- Wrestling (18)
8- Ice Hockey (39.3)	8- Skiing (17,113)	8- Skiing (815)	8- Canoe-Kayak (16)
9- Baseball/Softball (39)	9- Rowing (14,696)	9- Rowing (673)	9- Judo (15)
10- Aquatics (36)	10- Volleyball (13,401)	10- Volleyball (602)	9- Shooting (15)
11- Cycling (35)	11- Handball (12,141)	11- Ice Hockey (540)	11- Rowing (14)
12- Handball (30)	12- Ice Hockey (9,951)	12- Golf (491)	11- Weightlifting (14)
13- Table Tennis (27)	13- Triathlon (9,420)	13- Gymnastics (469)	13- Boxing (13)
14- Judo (25)	14- Gymnastics (9,049)	14- Handball (440)	14- Fencing (12)
15- Badminton (24.9)	15- Golf (8,980)	15- Triathlon (425)	15- Biathlon (11)
16- Wrestling (24)	16- Wrestling (7,725)	16- Wrestling (400)	16- Sailing (10)
17- Weightlifting (20)	17- Sport Climbing (6,581)	17- Sport Climbing (338)	17- Taekwondo (8)
18- Skiing (19)	18- Judo (5,941)	18- Skating (334)	17- Karate (8)
19- Shooting (18)	19- Weightlifting (5,476)	19- Weightlifting (264)	19- Equestrian (6)
20- Gymnastics (17)	20- Skating (4,915)	20- Judo (261)	20- Tennis (5)
21- Fencing (16.5)	21- Boxing (3,403)	21- Boxing (223)	20- Badminton (5)
22- Archery (16.4)	22- Field Hockey (3,247)	22- Canoe-Kayak (180)	20- Table Tennis (5)
23- Rugby Sevens (15.7)	23- Taekwondo (3,224)	23- Field Hockey (167)	20- Archery (5)
24- Taekwondo (15.4)	24- Canoe-Kayak (2,914)	24- Taekwondo (159)	24- Basketball (4)
25- Sailing 15	25- Karate (2,395)	25- Badminton (143)	24- Volleyball (4)
26- Skating (14)	26- Badminton (2,265)	26- Karate (113)	24- Bobsleigh (4)
27- Karate (13)	27- Sailing (1,535)	27- Sailing (109)	24- Skateboarding (4)
28- Rowing (12.2)	28- Rugby Sevens (1,411)	28- Surfing (100)	24- Luge (4)
29- Triathlon (12.1)	29- Shooting (1,247)	29- Fencing (90)	29- Triathlon (3)
30- Field Hockey (12)	30- Fencing (1,170)	29- Table Tennis (90)	29- Curling (3)
31- Equestrian (11.5)	31- Table Tennis (1,125)	31- Rugby Sevens (89)	31- Football (2)
32- Curling (10.6)	32- Surfing (949)	32- Shooting (55)	31- Baseball (2)
33- Biathlon (10.5)	33- Equestrian (720)	33- Equestrian (52)	31- Handball (2)
34- Canoe-Kayak (9.3)	34- Biathlon (710)	34- Biathlon (47)	31- Ice Hockey (2)
35- Modern Pentathlon (6.7)	35- Archery (591)	35- Archery (43)	31- Golf (2)
36- Surfing (4.7)	36- Bobsleigh (298)	36- Skateboarding (27)	31- Sport Climbing (2)
37- Bobsleigh (4.4)	37- Skateboarding (286)	37- Bobsleigh (19)	31- Field Hockey (2)
38- Luge (4)	38- Modern Pentathlon (73)	38- Modern Pentathlon (12)	31- Rugby Sevens (2)
39- Skateboarding (3)	39- Luge (59)	39- Curling (8)	31- Surfing (2)
40- Sport Climbing (2.2)	40- Curling (25)	40- Luge (6)	31- Modern Pentathlon (2)

Tables 10, 11 will show the results obtained:

**Table 10:** Correlations between the number of citations, the sports level of competition and the number of medals at the olympics.

R and P values	Correlation of sports number of citations/sports level of competition	Correlation of sports number of citations/sports number of medals	Correlation of sports level of competition/sports number of medals
R	0.74	0.27	0.01
P	<0.001	NS	NS

**Table 11:** Correlations between the number of articles, the sports’ level of competition and the number of medals at the olympics.

R and P values	Correlation of sports number of articles/sports level of competition	Correlation of sports number of articles/sports number of medals	Correlation of sports level of competition/sports number of medals
R	0.70	0.37	0.01
P	<0.001	<0.05	NS

Tables 10, 11 show that there are significant correlations between the sports level of competition, the number of citations and the articles. On the other hand, the correlations between the number of medals offered and the number of citations and articles are weak. And finally, the level of competition and the number of medals offered are not related.

Table 9 actually allows us to see pertinent examples of the strong convergences between the sports level of competition, the number of citations and the articles. Indeed, 70% of the 10 most competitive sports are among the 10 sports with the highest number of articles and citations: football, basketball, tennis, athletics, volleyball, baseball/softball, and aquatics. Concerning the 10 less competitive sports, 8 of them (80%) are among the 10 less cited sports (equestrian, curling, biathlon, modern pentathlon, surfing, bobsleigh, luge, and skateboarding), and 7 of them are among the 10 sports with the lowest number of articles (equestrian, curling, biathlon, modern pentathlon, bobsleigh, luge, and skateboarding). Since the level of competition of each sport is the sum of its popularity and universality, this shows the significant convergence between the economic and scientific sizes of each sport.

These economic and scientific weights are, on the other hand, not considered at all in the OMT: only 4 of the top 10 sports (40%) offering the highest number of medals are among the 10 sports with the highest number of articles and citations: skiing, aquatics, athletics, and cycling, with only 2 of them (20%) are among the 10 most competitive sports: athletics and aquatics. Football, the most competitive and cited sport in the world, belongs to the 10 sports offering the lowest number of medals. The coefficient for football in terms of level of competition is 6.5 times higher than the one for skiing, the number one sport in terms of number of medals offered: skiing can provide 55 medals, 27.5 times more than football, which can only give two medals per country. Football has 6 times more scientific articles than skiing and is cited 9 times more.

In the bottom 10 of the sports offering the highest number of medals, only 2 (20%) belong to the bottom 10 in terms of number of citations (surfing and modern pentathlon) and also 2 (20%) in the total number of articles (rugby sevens and modern pentathlon). Three (30%) of these “least medals sports” belong to the bottom 10 when it comes to the level of competition: sport climbing, surfing, and modern pentathlon. 50% of the 10 most competitive and cited sports (football, basketball, tennis, volleyball, and baseball/softball), which are also in the top 10 in number of articles, belong to the bottom half of the sports offering the highest number of medals. The total number of medals that these five sports offered all together in the 2020 Olympics is 17, which represents only 60% of the number of medals offered by skating alone (28 medals), the 27th in terms of level of competition, 20th in the number of citations, and 18th in the number of scientific papers. If we add the level of competition coefficients of these 5 sports, we reach an amount 26.5 times higher than the one of skating, and if we do the same calculi for the number of citations, the amount will be 50 times higher. As for their total number of scientific papers, the amount of these 5 sports will be 26 times higher than the skating one.

## Discussion

When asked about the WRCES (31), Danyel Reiche, author of the book “Success and Failure of Countries at the Olympics (32),” has advocated the idea that “Olympic medal counts have several weaknesses”, one of them being that “all medals are counted alike, regardless of the popularity of a sport”. For Reiche, the WRCES incorporates factors that “capture a more holistic view of measuring success in sports” and “should serve as a blueprint for a much-needed debate on Olympic medal count reform.”

For Brian Minikin (31), former regional manager in the Oceania National Olympic Committee, the “dogmatic adherence to the Olympic Games medal tally” has “led funds to be directed away from the establishment of viable and sustainable sport systems, in sports that are popular to play and watched in many countries.” Minikin believes that the WRCES “offers hope that there is a better way.”

It is, therefore, with a reformist scientific approach that the WRCES was created to allow for an accurate measurement of the countries’ success in elite sport, and the level of competition of each sport. The purpose of this paper was to strengthen the scientific validity of the WRCES by correlating it with the bibliometric research published by Millet et al. (6), which has led to obtain the following important findings:

- The comparative analysis between the level of competition, scientific size and number of medals offered has shown that the Olympic medal counts does not reflect the global representativeness of the different Olympic sports.
- The inaccuracies of the OMT call for an awareness of the different stakeholders of the international sport movement, National Olympic Committees (NOCs), National Sport Governing Bodies (NSGB), and International Federations (IFs) to consider a scientific index that will accurately measure the countries performances in elite sport, as well as the level of representativeness of the recognized sports. In this aspect, the WRCES has shown to be a very solid alternative.
- With the Olympics being the flagship competition of international sport, sustaining the WRCES and the sports bibliometrics is a key factor to benchmark the global representativeness of the Olympic sports, and to propose to countries a measurement tool they can use to elaborate sustainable and holistic national sports policies. It can also provide for the IFs, which disciplines are part of the Olympic program, a scientific evaluation of the international development of their sports, whether it is related to their universality, global media popularity, level of competition, and references in the scientific community. In that perspective, these IFs can use the data presented by the WRCES and the work done by Millet et al. (6) to implement strategies that will lead to the growth of their sports.

The WRCES has also allowed the creation of three other research-based sports indexes for countries:

- The WRCES Merit Ranking, which rewards countries whose performance exceeds their economic capacities (33). This ranking was calculated by carrying out correlations between the results of WRCES, population and GDP over six consecutive years: 2014, 2015, 2016, 2017, 2018, and 2019. The calculations showed that with a correlation lower than 0.4, population has no impact on the sporting performance of nations, whereas with a correlation higher than 0.7, GDP is an essential factor. Therefore, the WRCES Merit Ranking is calculated by doing the difference between the WRCES ranking and the GDP ranking of each country. The higher the difference in favor of WRCES, the higher the country's score in the WRCES Merit Ranking will be.
- The World's Fittest Countries Ranking (WFCR), which integrates three variables: countries’ participations in sport, which is measured by the rate of sports in which each country is ranked in the WRCES independently from its results, the Obesity Rate (OR), and the Prevalence of Undernourishment (PoU). The aim of the WFCR is to provide different governments with the information needed to develop national policies targeting the improvement of the fitness levels of their citizens. The WFCR is recognized by the International Federation of Physical Education and Sports (FIEPS) (4), an organization including 142 countries and recognized by the IOC, the United Nations, Educational, Scientific and Cultural Organization » (UNESCO), and the International Council of Sport Science and Physical Education (ICSSPE) (34).
- The World Sport Power Index (WSPI), which measures countries’ capacities to use sport to have a geopolitical impact (35). The WSPI is made up of three variables: the performance of national teams indicated by the WRCES, the global media popularity of the local professional leagues, and the capacity of countries to organize major sporting events.

With the WRCES and the three other indexes which arise from it, countries’ governments will have indicators that they can use to set up policies aiming for success in elite sport, the development of physical activity within the population, and the strengthening of geopolitical impact through sport. The objective of this research was therefore to reinforce the scientific validity of the WRCES, with the aim of contributing to future research in the academic field of sport policy.

This paper can also not omit the limitations of the WRCES, which should be addressed in future research. The current methodology implies that the universality coefficient of each sport is calculated based on the number of national federations, its presence in the programs of the Olympics, ISF, FISU, CISM, USIP, IMGA, WTGF, SO, CISS, CSIT, and CICG. This variable could be improved by having more accurate data on the number of registered athletes and competitors.

The media popularity component could also be ameliorated. As it is, it considers the GDP of every country, which is pertinent to relate to the wealth of each state. However, more accurate indicators evaluating the economic size of each country's sport industry will allow to know with more exactitude how much funding the different sports are generating. It can also consider more holistic indicators such as the Human Development Index (36), which includes the health, income, education and living conditions in each country

## Data Availability

All the data can be found in the official website of the International Center for Sport Policy & Governance (ICSPG, https://www.ndu.edu.lb/international-center-for-sport-policy-and-governance/home). Further inquiries can be directed to the corresponding author.

## Footnotes

^1^The portal Alexa has ceased to exist in 2023. Since this year, the ICSPG uses the portal “Semrush”.

^2^The WRCES considers all the sports recognized by SportAccord (https://www.sportaccord.sport/what-is-sportaccord/, retrieved in July 2025), Olympics and non-Olympics, plus others not yet recognized, but having a significant media popularity. For the year 2019, 112 sports were considered.

^3^the WRCES usually calculates the performances of all the countries in all the sports recognized by SportAccord, Olympics, and non-Olympics. Only the WRCES coefficients of the Olympic sports will be considered in this study.

## References

[1] International Olympic Committee. “Olympic Charter”. Lausanne: International Olympic Committee (2020). art. 57.

[2] Nassif N Raspaud M. Correlations between sport results, population, GDP, area, and research rankings. In: Nassif N Raspaud M, editors. National Success in Elite Sport: Exploring the Factors That Lead to Success. Cham: Springer Nature Switzerland (2023). p. 69–75.

[3] Nassif N. World ranking of countries in elite sport. Riv Diritto Econ Dello Sport. (2018) 14(57):55–75.

[4] Official website of the International Center for Sport Policy & Governance (ICSPG) (2024). Available online at: https://www.ndu.edu.lb/international-center-for-sport-policy-and-governance/home (Accessed July 10, 2024).

[5] Nassif N Raspaud M. Measurement of Countries’ performances and successes in elite sport: the world ranking of countries in elite sport. In: Nassif N Raspaud M, editors. National Success in Elite Sport: Exploring the Factors That Lead to Success. Cham: Springer Nature Switzerland (2023). p. 1–32.

[6] Millet GP Brocherie F Burtscher J. Olympic Sports science—bibliometric analysis of all summer and winter Olympic sports research. Front Sports Act Living. (2021) 3:772140. 10.3389/fspor.2021.772140 34746779 PMC8564375

[7] Official website of the International Olympic Committee (2024). Available online at: https://olympics.com/en/paris-2024/medals (Accessed August 1, 2024).

[8] Official website of the Entertainment and Sports Programming Network (ESPN), Paris Games Medal Tracker (2024). Available online at: https://www.espn.com/olympics/summer/2024/medals (Accessed August 1, 2024).

[9] De Bosscher V. The Global Sporting Arms Race: An International Comparative Study on Sports Policy Factors Leading to International Sporting Success. Aachen: Meyer & Meyer Verlag (2008).

[10] Official website of the International Olympic Committee (2024). Available online at: https://olympics.com/ioc/faq/sports-programme-and-results/the-olympic-programme-comprises-sports-disciplines-and-events-what-is-the-difference-between-the-three (Accessed August 1, 2024).

[11] Official website of the National Broadcasting Channel (NBC). (2024). Available online at: https://www.nbcphiladelphia.com/paris-2024-summer-olympics/olympic-medals-per-capita-2024/3933801/ (Accessed August 14, 2024).

[12] Official website of Radio New Zealand (RNZ). (2024). Available online at: https://www.rnz.co.nz/news/media-technology/524896/the-kiwi-brain-behind-the-paris-olympic-s-medal-per-capita-tally (Accessed August 14, 2024).

[13] Official website of The Sydney Morning Herald (2024). Available online at: https://www.smh.com.au/sport/the-countries-that-have-won-most-olympic-medals-per-head-of-population-20240811-p5k1dw.html (Accessed August 14, 2024).

[14] Bernard AB Busse MR. Who wins the olympic games: economic resources and medal totals. Rev Econ Stat. (2004) 86(1):413–7. 10.1162/003465304774201824

[15] Butter D A F Van Der Tak CM. Olympic medals as an indicator of social welfare. Soc Indic Res. (1995) 35(1):27–37. 10.1007/BF01079236

[16] Kuper GH Sterken E. Olympic Participation and Performance Since 1896. Groningen: Center for Economic Research - University of Groningen (2001).

[17] Bian X. Predicting Olympic medal counts: the effects of economic development on Olympic performance. Park Place Econo. (2005) 13(1):37–44.

[18] De Bosscher V Shibli S Weber AC. Is prioritisation of funding in elite sport effective? An analysis of the investment strategies in 16 countries. Eur Sport Manag Quart. (2019) 19(2):221–43. 10.1080/16184742.2018.1505926

[19] Henry I Dowling M Ko LM Brown P. Challenging the new orthodoxy: a critique of SPLISS and variable-oriented approaches to comparing sporting nations. Eur Sport Manag Quart. (2020) 20(4):520–36. 10.1080/16184742.2020.1719428

[20] Nassif N Raspaud M. National Success in Elite Sport. Cham: Springer Books (2023).

[21] Hanief YN. Bibliometric analysis of sports studies in the “Journal Sport Area”. J Sport Area. (2021) 6(2):263–74. 10.25299/sportarea.2021.vol6(2).6845

[22] Gholampour S Noruzi A Gholampour B Elahi A. Research trends and bibliometric analysis of a journal: sport management review. Webology. (2019) 16(2):223–41. 10.14704/WEB/V16I2/a200

[23] López-Carril S Escamilla-Fajardo P González-Serrano MH Ratten V González-García RJ. The rise of social media in sport: a bibliometric analysis. Int J Innov Technol Manag. (2020) 17(06):2050041. 10.1142/s0219877020500418

[24] Escher IWONA. Sustainable development in sport as a research field: a bibliometric analysis. J Phys Educ Sport. (2020) 20(5):2803–12. 10.7752/jpes.2020.s5381

[25] Ciomaga B. Sport management: a bibliometric study on central themes and trends. Eur Sport Manag Quart. (2013) 13(5):557–78. 10.1080/16184742.2013.838283

[26] Baier-Fuentes H González-Serrano MH Alonso-Dos Santos M Inzunza-Mendoza W Pozo-Estrada V. Emotions and sport management: a bibliometric overview. Front Psychol. (2020) 11:1512. 10.3389/fpsyg.2020.01512 32754088 PMC7366861

[27] Shilbury D. A bibliometric analysis of four sport management journals. Sport Manag Rev. (2011) 14(4):434–52. 10.1016/j.smr.2010.11.005

[28] Belfiore P Ascione A Di Palma D. Technology and sport for health promotion: a bibliometric analysis. J Human Sport Exerc. (2019) 15(4):932–42. 10.14198/jhse.2020.154.19

[29] González-Serrano MH Jones P Llanos-Contrera O. An overview of sport entrepreneurship field: a bibliometric analysis of the articles published in the web of science. Sport Soc. (2020) 23(2):296–314. 10.1080/17430437.2019.1607307

[30] Pilon F Prince F. Does producing scientific articles lead to paralympic podiums? Biomechanics. (2024) 4(1):123–43. 10.3390/biomechanics4010008

[31] Official website of the company World Sports Rankings (WSR) (2024). Available online at: https://sportsrankings.world/ (Accessed August 28, 2024).

[32] Reiche D. Success and Failure of Countries at the Olympic Games. Milton Park: Routledge (2016).

[33] Nassif N Keyrouz K. Creating a global Index measuring Countries’ levels of fitness: the “world’s fittest countries ranking”. In: Antala B Labudová J Kaplánová A van Heel J Novak D Wang X, editors. Physical Education and Sport for Children, Youth and Adults and Healthy Active Living Researches—Best Practices—situation. Slovakia: International Federation of Physical Education (2022). p. 265–76.

[34] Official website of the International Federation of Physical Education and Sports (FIEPS) (2024). Available online at: https://www.fiepseurope.com/ (Accessed August 28, 2024).

[35] Nassif N. The world sport power index: measuring States’ capacities to use sport as an instrument of soft power. Siyasat Arabiya. (2022) 10(57):46–57. 10.31430//OXAQ6535

[36] Official website of the UNDP (2025). Available online at: https://hdr.undp.org/data-center/human-development-index#/indicies/HDI (Accessed July 18, 2025).

[37] Official website of SportAccord (2025). Available online at: https://www.sportaccord.sport/what-is-sportaccord/ (Accessed July 18, 2025).

